# Editorial: Advances in computer vision: from deep learning models to practical applications

**DOI:** 10.3389/fnins.2025.1615276

**Published:** 2025-05-09

**Authors:** Hancheng Zhu, Rui Yao, Lu Tang

**Affiliations:** ^1^School of Computer Science and Technology, China University of Mining and Technology, Xuzhou, China; ^2^Xuzhou Medical University, Xuzhou, China

**Keywords:** computer vision, deep learning models, practical applications, lightweight network, medical image analysis and processing system

Computer vision has emerged as one of the most transformative areas of artificial intelligence, with deep learning models driving unprecedented advancements in both theoretical understanding and practical applications. Over the past decade, the rapid development of deep learning techniques has enabled machines to perform tasks such as image recognition, object detection, and video analysis with remarkable accuracy and efficiency. However, as the field continues to evolve, there is a growing need to bridge the gap between theoretical models and real-world applications to ensure that these technologies are powerful but also practical, efficient, and scalable. This Research Topic, “*Advances in computer vision: from deep learning models to practical applications*,” is dedicated to exploring the latest innovations in computer vision that are addressing these challenges and pushing the boundaries of what is achievable.

The articles in this Research Topic represent a diverse range of research directions and applications, reflecting the interdisciplinary nature of computer vision. From efficient single-image super-resolution techniques to lightweight network architectures for traffic sign recognition, and from medical image processing to action recognition in autonomous systems, the contributions highlight the versatility, and potential of computer vision technologies. Below, we provide a brief overview of the accepted articles, emphasizing their key contributions and practical implications.

## Efficient and lightweight deep learning models for real-world applications

One of the central themes of this Research Topic is the development of efficient and lightweight deep learning models that can operate effectively in resource-constrained environments. An et al. presented a lightweight network architecture based on an enhanced LeNet-5 model for traffic sign recognition. By optimizing the network structure and reducing the number of parameters, they achieved state-of-the-art performance on standard benchmarks, making their solution suitable for deployment in real-world autonomous driving systems.

Qu and Ke proposed an asymmetric large kernel distillation network for single image super-resolution, which leveraged asymmetric kernels to achieve high computational efficiency while maintaining superior performance in image restoration. Their approach demonstrated the importance of balancing model complexity and practical applicability, particularly in scenarios where computational resources are limited.

Xie et al. proposed an extremely lightweight pathological myopia instance segmentation method (SMLS-YOLO) that combined attention mechanisms with efficient network design to achieve real-time performance. Their approach was particularly valuable for applications in ophthalmology, where rapid and accurate segmentation is critical for diagnosing and monitoring conditions such as pathological myopia. The integration of attention mechanisms into lightweight models highlighted the importance of optimizing both computational efficiency and accuracy to ensure that these technologies can be deployed in real-world settings.

## Deep learning in medical image processing

Medical image processing is another area where deep learning has shown tremendous potential. Li L. et al. explored the use of Swin Transformer-based automatic delineation of the hippocampus in MRI scans for hippocampus-sparing whole-brain radiotherapy. Their work showcased the effectiveness of transformer-based architectures in medical image segmentation, providing a more accurate and automated approach to treatment planning.

Tian and Zhang presented a GAN-guided nuance perceptual attention network (G2NPAN) for multimodal medical fusion image quality assessment. Their work combined generative adversarial networks (GANs) with attention mechanisms to evaluate the quality of fused medical images, ensuring that the outputs were both visually appealing and diagnostically useful. This approach highlighted the importance of integrating advanced deep learning techniques with practical applications in healthcare, where image quality and interpretability are critical.

Zhou et al. focused on lymph node segmentation in lung cancer diagnosis, introducing a dual-stream feature-fusion attention U-Net (DFA-UNet). By incorporating attention mechanisms into the U-Net architecture, they achieved improved segmentation accuracy and computational efficiency, which are essential for clinical applications where time and resource constraints are significant.

Gu et al. proposed a motion-sensitive network for action recognition in autonomous systems, leveraging insights from motion perception to improve decision-making in real-time control scenarios. Their work underscored the importance of designing models that can efficiently process dynamic visual inputs, which have direct applications in robotics and autonomous vehicles.

## Practical applications and beyond

The practical applications of computer vision are vast and varied, ranging from culture to education to security systems. Ramesh et al. presented a hybrid manifold smoothing and label propagation technique for handwritten Kannada character recognition, demonstrating how advanced deep learning methods can be adapted for tasks involving handwritten text. Their work had implications for document analysis, OCR systems, and cultural heritage preservation, where handwritten text recognition remains a challenging yet important task.

Li X. et al. proposed a parameter-dense three-dimensional convolution residual network for classroom teaching applications. By incorporating dense 3D convolutions, they demonstrated improved performance in handling complex, multidimensional data.

The work by Wang on attention-enhanced computation in multimedia affective computing explored how attention mechanisms can be used to evaluate and analyze visual perception, particularly in the context of affective computing. By integrating attention-based models, Wang demonstrated how visual perception can be quantified and optimized for applications such as emotion recognition and human-computer interaction.

Ma et al. proposed a study on face anti-spoofing based on pseudo-negative feature generation, addressing the critical issue of ensuring security and reliability in facial recognition systems. By generating pseudo-negative features to enhance robustness against spoofing attacks, their work contributed to the development of more secure and trustworthy biometric systems. This is particularly relevant in practical applications where facial recognition is increasingly used for authentication and access control.

This Research Topic, “*Advances in computer vision: from deep learning models to practical applications*,” reflects the current state of the field and its trajectory toward solving real-world problems. Collectively, the articles demonstrate how deep learning models can be optimized for efficiency, scalability, and practicality while maintaining high performance in diverse applications. From lightweight architectures for traffic sign recognition and super-resolution imaging to advanced attention mechanisms for medical image segmentation and action recognition, these contributions highlight the interdisciplinary nature of computer vision and its potential to revolutionize diverse domains.

This Research Topic of articles inspires further research and collaboration between researchers, practitioners, and industry. The integration of deep learning with practical applications not only enhances the functionality of computer vision systems but also ensures their relevance and usability in addressing real-world challenges. As the field continues to grow, we anticipate even more exciting developments that will bridge the gap between theoretical models and practical deployment, paving the way for smarter, more efficient, and more reliable vision technologies.

Finally, we would like to extend our gratitude to all the authors for their insightful contributions and to the reviewers for their valuable feedback. This Research Topic will serve as a valuable resource for researchers and practitioners alike, fostering innovation and advancing the field of computer vision toward practical and impactful applications.

